# A weighted and integrated drug-target interactome: drug repurposing for schizophrenia as a use case

**DOI:** 10.1186/1752-0509-9-S4-S2

**Published:** 2015-06-11

**Authors:** Liang-Chin Huang, Ergin Soysal, W Jim Zheng, Zhongming Zhao, Hua Xu, Jingchun Sun

**Affiliations:** 1 School of Biomedical Informatics, The University of Texas Health Science Center at Houston, Houston, TX 77030, USA; 2 Departments of Biomedical Informatics and Cancer Biology, Vanderbilt University School of Medicine, Nashville, TN 37203, USA

## Abstract

**Background:**

Computational pharmacology can uniquely address some issues in the process of drug development by providing a macroscopic view and a deeper understanding of drug action. Specifically, network-assisted approach is promising for the inference of drug repurposing. However, the drug-target associations coming from different sources and various assays have much noise, leading to an inflation of the inference errors. To reduce the inference errors, it is necessary and critical to create a comprehensive and weighted data set of drug-target associations.

**Results:**

In this study, we created a weighted and integrated drug-target interactome (WinDTome) to provide a comprehensive resource of drug-target associations for computational pharmacology. We first collected drug-target interactions from six commonly used drug-target centered data sources including DrugBank, KEGG, TTD, MATADOR, PDSP K_i _Database, and BindingDB. Then, we employed the record linkage method to normalize drugs and targets to the unique identifiers by utilizing the public data sources including PubChem, Entrez Gene, and UniProt. To assess the reliability of the drug-target associations, we assigned two scores (Score_S and Score_R) to each drug-target association based on their data sources and publication references. Consequently, the WinDTome contains 546,196 drug-target associations among 303,018 compounds and 4,113 genes. To assess the application of the WinDTome, we designed a network-based approach for drug repurposing using mental disorder schizophrenia (SCZ) as a case. Starting from 41 known SCZ drugs and their targets, we inferred a total of 264 potential SCZ drugs through the associations of drug-target with Score_S higher than two in WinDTome and human protein-protein interactions. Among the 264 SCZ-related drugs, 39 drugs have been investigated in clinical trials for SCZ treatment and 74 drugs for the treatment of other mental disorders, respectively. Compared with the results using other Score_S cutoff values, single data source, or the data from STITCH, the inference of 264 SCZ-related drugs had the highest performance.

**Conclusions:**

The WinDTome generated in this study contains comprehensive drug-target associations with confidence scores. Its application to the SCZ drug repurposing demonstrated that the WinDTome is promising to serve as a useful resource for drug repurposing.

## Background

Computational pharmacology plays an important role in the drug development, given that the traditional approaches have the low success rate and the increasing cost [1,2]. It provides a macroscopic view and a deeper understanding of the molecular mechanisms of drug action by integrating multiple data sets through a variety of informatics approaches [3]. Among these approaches, network-assisted method provides a unique platform by interrogating the relationships among drugs, proteins, and diseases to predict drug repurposing [3,4]. The drug repurposing (also called repositioning or re-profiling) is a process to identify novel indication for already existing drugs [5]. It is an essential strategy for drug development due to its capability of identifying novel indications of an approved drug, which in turn accelerates the drug development process [5,6]. Therefore, some studies have applied the computational approaches to drug repurposing based on drug-disease/side-effect association [7], genome-wide association studies (GWAS) [8], or gene expression profiles [9]. However, inferences of new drug indication heavily rely on the drug-target association (also called interactome). Therefore, reliable drug-target data are fundamental and crucial for supporting drug action inferences.

During the last decade, many effects involving a multitude of methods has been made to the determination of the interactions between drugs and their targets. A number of databases have been created to systematically store the drug-target interactions such as SuperTarget [10], Psychoactive Drug Screening Program (PDSP) [11], Drug Gene Interaction Database (DGIdb) [12], and STITCH ('search tool for interactions of chemicals') [13]. According to the nature of data generation, these existing drug-target databases can be categorized into three groups: expert-curated [10-12,14-20], predicted [12,17,21,22], and integrative [10,12,13,21-29]. The expert-curated drug-target associations are manually extracted from the literature, including the small-scale studies and high-throughput screening (HTS). The small-scale studies possess the highest reliability while the HTS studies have issues about systematic errors on quality control and hit selection [30]. Predicted drug-target associations are inferred from known drug targets or other drug actions. Although the reliability of this type of data is not comparable to that of the expert-curated associations, the predicted associations provide researchers potential drug targets or off-targets for further investigation [21]. The integrative databases consist of the expert-curated and predicted associations such as STITCH, whose quality relies on the original data sources and comprehensive evaluation of the data. Additionally, it is not easy to trace back to the original data and assess their reliability if the integrated database did not provide the original data sources. Moreover, addressing the issues of data redundancy and both drug and target identifiers' heterogeneities is another major problem during the data integration.

In this study, we integrated drug-target associations from six commonly used drug-target centered data sources and assigned a reliability assessment for each drug-target association to create one weighted and integrative drug-target interactome (WinDTome). In the process of building the WinDTome, we addressed the intrinsic issues of redundancy and heterogeneous identifiers by using the public drug-centered databases and gene-centered databases. The WinDTome will serve as a fundamental with assessments for the network-assisted computational pharmacology for drug repurposing. To illustrate its application, we used schizophrenia (SCZ) as an example. We started with the known medicines used to treat SCZ and SCZ candidate genes to predict the non-SCZ drugs that might have potentials for SCZ treatments based on the higher confident drug-target associations in WinDTome and protein-protein interactions. We inferred 264 drugs that might have potential to treat the SCZ. Among them, 39 drugs and 74 drugs have been investigated to treat SCZ and other mental disorders based on the clinical trial records in the database "clinical trials.gov", respectively. We further compared the performance of this process with the results based on other score cutoff values, single data sources, and the high confident data from STITCH. The comparative results showed that the WinDTome is promising for providing the fundamental of drug-target interactions for drug repurposing.

## Materials and methods

### Sources of drug-target interactome

We integrated drug-target interactions that were extracted from six public data sources. They were DrugBank [14], Kyoto Encyclopedia of Genes and Genomes (KEGG) Drug [24], Therapeutic Target Database (TTD) [23], Manually Annotated Targets and Drugs Online Resource (MATADOR) [10], NIMH Psychoactive Drug Screening Program (PDSP) K_i _Database [11], and BindingDB [15].

DrugBank is a freely available web-enable database that contains detailed drug data with comprehensive drug target and drug action information [14]. We downloaded the file "drugbank.xml" from the DrugBank website on February 2014. From the file, we obtained the drug information such as drug's status, descriptive indication, target's UniProt accession numbers (ACs), references, and external links to ChEBI [31], PubChem Compound (CID) and Substance (SID).

KEGG Drug is a comprehensive information resource for approved drugs in Japan, USA, and Europe [24]. We accessed the data of KEGG Drug through KEGG FTP server on May 2014, and extracted drugs, targets, and external links to DrugBank and PubChem Substance and the drug targets and corresponding genes' Entrez Gene IDs.

TTD is a database that contains the known and explored therapeutic protein and nucleic acid targets, the disease, pathway information and the corresponding drugs. The approved drugs, experimental drugs, and clinical trial drugs, and their primary targets were obtained from a comprehensive search of literatures and FDA labels [23]. The data set of TTD was downloaded on October 2013. Drug-target associations along with drug's status and structuralized indication are available in the file "TTD_download.txt". The drug's external links to ChEBI, PubChem Compound, and PubChem Substance were obtained from the file "TTD_crossmatching.txt". The target's UniProt ACs are available in the "TTD_uniprot_all.txt" file.

MATADOR is a resource for direct and indirect protein-chemical interactions that was assembled by automated text mining followed by manual curation. Each interaction contains links to PubMed abstracts or OMIM entries that were used to deduce the interaction [10]. The data set of MATADOR was on March 2007. Drug-target associations, including both direct and indirect interactions, are available in the "matador.tsv" file. PubChem Compound CID and UniProt AC were utilized to represent a drug's identifier and a target's identifier, respectively.

The PDSP K_i _database is a repository for numerous drugs' and drug candidates' affinity (*i.e*., K_i _values) information [11]. The data was downloaded on January 2011. We obtained the drug-target associations along with their references, drug's PubChem CID, and gene symbols from the "kidb110121.txt" file.

The BindingDB a public, web-accessible database of measured binding affinities, focusing chiefly on the interactions of protein considered to be drug targets with small, drug-like molecules [15]. We downloaded the data on June 2014. We extracted drug-target associations and references from the file "BindingDB_All.tsv", in which UniProt AC was used to represent the target's identifier. We also extracted the drugs' external links to DrugBank, ChEBI, PubChem Compound and Substance from the TSV file.

### Drug and target name normalization

We created the WinDTome that consists of the drug-target associations from six databases. To normalize drug and target names from the six sources, we utilized the drug information from PubChem and gene/protein information from Entrez Gene and UniProt. PubChem is an open and comprehensive database with information on the biological activities of small molecules collected from 276 data sources. It contains three linked databases: Substances, Compounds, and Bioassay [32]. In this study, we utilized the Compound CIDs and their synonyms as a major part of drug normalization. We first downloaded the "CID-Synonym-filtered" file from PubChem on April 2014. If external links for PubChem Compound in the original data sources are not available, we utilized the string exact matching approach to link the drug's names to PubChem CIDs by the synonyms. For those cannot match to PubChem CID, we kept the original names from their corresponding data sources. We assigned a unified identifier (UID) to each drug and also kept the five identifiers including drug name, DrugBank ID, PubChem CID, PubChem Substance ID (SID), ChEBI ID.

In this study, we utilized the gene symbols to represent the drug targets by matching the gene ID and symbol to UniProt ACs. The human gene information and annotation are available in the "gene_info" file, which was downloaded from NCBI Gene on May 2014 [33]. The file provides gene's ID, name, symbol, and taxonomy information. We utilized the gene ID, name, and symbol to present the drug target and gene taxonomy to filter out the targets that not belong to human. The UniProt is built as a depository of protein knowledge including sequence and functional information [34]. The data set of UniProt is available at http://www.uniprot.org/downloads (accessed May 2014). The mapping file "idmapping.dat" was served as a linkage between UniProt AC and Entrez Gene ID, and it helps to normalize heterogeneous target name or identifier in different drug target databases to Entrez Gene ID.

### Drug and target classification

For further exploring the drug and target classification, we explored the Anatomical Therapeutic Chemical (ATC) code and Protein Analysis Through Evolutionary Relationships (PANTHER) protein class tool to classify the normalized drugs and targets, respectively. The ATC Classification System, controlled by the World Health Organization Collaborating Centre for Drug Statistics Methodology (WHOCC), is used for the classification of drugs. This pharmaceutical coding system hierarchically categorizes drugs into different groups based on the drugs' therapeutic and chemical properties. In this study, the drug's ATC code is obtained from both DrugBank and KEGG. PANTHER is a comprehensive, curated database of protein families [35]. The analysis was performed on January 2014. The UniProt ACs used in PANTHER as protein IDs were converted to Entrez Gene IDs for being compatible with the unified target ID in WinDTome. In order to simplify the drug targets' protein classes, we assigned the "protein class" as level 1 class. In the study, we utilized level 2 classes.

### Scoring system

We designed two scoring systems to assess the reliability of the drug-target associations in the WinDTome: Score_S and Score_R. For each drug-target pair, the Score_S is its frequency of the presence in the six sources. If a drug-target association exists in all the six sources, its Score_S will be 6, and it was considered a highest reliable association.

As discussed in the introduction section, some drug-target associations might originated from HTS studies, which have the limitation of the specificity and accuracy in drug-target associations. To distinguish the HTS studies from small-scale studies needs a large amount of manual checking, which requires extensive time for domain experts to accomplish it. In order to address this issue, we developed a Score_R to assess a drug-target association's reliability based on the number of references and the reference's specificity. For each drug-target association (*x*), Score_R was calculated by the following equation:

$$S_{R}\left(x\right)=\overset{n}{\underset{i=1}{\sum }}\frac{d_{r_{i}}\cdot t_{r_{i}}}{f_{r_{i}}^{2}}$$

*n *is the number of PubMed references supporting the association *x; r_i _*represents the *i*th reference supporting the association *x; f_r _*is the number of drug-target associations supported by the reference *r; d_ri _*and *t_ri _*represent the numbers of drugs and targets reported in the reference *r*, respectively. An HTS study tends to have either high *d *and low *t *or low *d *and high *t*. Thus, given that two references have the same frequency in entire the data set, the reference of HTS study would have a lower score than the specific drug-target study does. Moreover, Score_R was also constructed of an intuitive assumption: the more references, the higher score. Thus, Score_R was a score of an accumulation of all the specificity scores of the references related to a drug-target association. The higher Score_R of a drug-target association represents the higher reliability.

### Network-based inference of potential drugs to treat SCZ

To demonstrate the application of WinDTome for drug repurposing, we inferred the drugs that have not yet been approved for SCZ treatment (non-SCZ drugs) as potential SCZ drugs based on the drugs used to treat SCZ (SCZ drugs) and their known targets. The rationale of this inference was based on the assumption that drugs having same targets with SCZ drugs, or drugs whose targets have direct interactions with SCZ drug target, might be potential SCZ drugs. In our previous study, we collected 32 SCZ drugs [36]. In this study, we updated the drug list by manually checking the drugs' indication field in the DrugBank. For each drug, if the field contains any keyword of "schizophrenia," "schizophrenic," "schizotypy," or "schizotypal," it may be a known SCZ drug. After manually check, we obtained 41 SCZ drugs. Then we obtained their targets from the DrugBank as known SCZ drug targets.

Starting the known SCZ drug targets and drug-target associations from WinDTome, we first obtained a group of non-SCZ drugs having associations with SCZ drugs' targets. Secondly, from drug-target associations from WinDTome, we obtained another group of non-SCZ drugs by requiring their target proteins are encoded by SCZ candidate genes [37] and these target proteins have interactions with known SCZ drug target proteins. The protein-protein interactions used here were obtained from the Protein Interaction Network Analysis (PINA) [38] (accessed in December 2012). During the two processes, we further required the drug-target associations with Score_S ≥ 3 in WinDTome.

### SCZ-relevant clinical trials

To evaluate the potential of the drugs that we inferred to treat SCZ, we exploited the SCZ-relevant clinical trials collected in the ClinicalTrials.gov (https://clinicaltrials.gov/). The ClinicalTrials.gov is a web-based resource that provides the information of publically and privately supported clinical studies on a wide range of diseases and conditions. To identify evidences of existing SCZ-relevant clinical trials to support the predictive potential SCZ drugs, we searched the drug names against ClinicalTrials.gov by setting the "Conditions" as "schizophrenia" and the "Interventions" as the name of each potential SCZ drug.

## Results

### Overview of the normalized drug-target interactome

**Figure 1:**
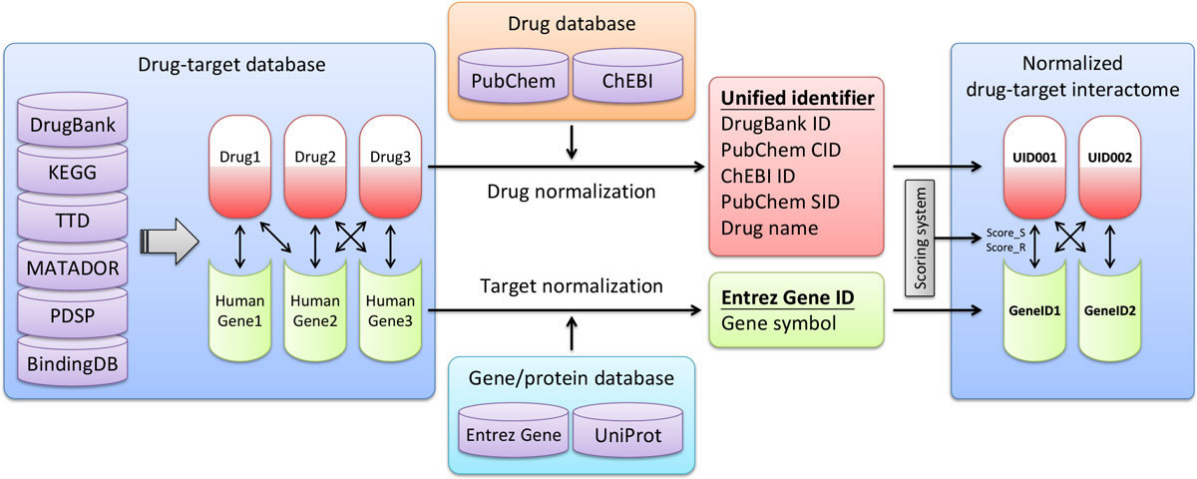
**The generation of the weighted and integrated drug target interactome (WinDTome)**. The processes of building WinDTome included three steps: data extraction and pre-processing (left); drug and target name normalization; and integration (right). The name normalization is the process to convert heterogeneous identifiers into one unified identifier. In this study, we utilized external data sources to convert heterogeneous drug and target identifiers into unified drug and target identifiers, respectively.

After extracting drug-target interactome from the six databases, we normalized drug names and target names for uniting identifiers and Entrez Gene IDs, respectively (Figure 1). In this process, heterogeneous identifiers of drugs and targets were mapped to the unified identifier systems (see Materials and Methods) to reduce the redundant drug-target associations among these sources. To further purify the data of WinDTome, we removed the drug-target associations in which 1) the length of the drug name is less than three, 2) the drug name is a numerical digit, and 3) the target does not belong to the human. Thus, we obtained in a total of 546,196 drug-target associations composing of 303,018 drugs and 4,113 human targets (Supplementary Table S1, Additional file 1). We further summarized the number of drugs, targets, and interactions for each database and their overlap proportion between any two databases (Table 1). We defined the proportion as the number of intersections divided by the smaller number of the two sources to represent the coverage of the overlap in the smaller data source. Among the 15 values of overlap proportion among the six databases, only three of the drug and target overlaps were much than 50%, and no values of the interactions were much than 50%. The results showed that, though some duplicated drug-target associations existed among the six drug-target databases, most of them had the lower overlap proportions. The observation indicated that these six sources have diverse types of drug-target association, which may be complementary to each other. Among these drugs, 1,572 were approved drugs, according to DrugBank and TTD. They had 3,041 targets and formed 22,059 drug-target associations.

**Table 1:** Summary of the drugs, targets, and their interactions in six data sources and their overlap

		Overlap (number^a^/proportion^b^)					
		
	Source	DrugBank	KEGG	TTD	MATADOR	PDSP	BindingDB
Drug	DrugBank	**4,316**	0.679	0.369	0.590	0.045	0.269
	KEGG	761	**1,121**	0.467	0.550	0.135	0.231
	TTD	1,594	524	**14,073**	0.405	0.069	0.425
	MATADOR	447	417	307	**758**	0.199	0.170
	PDSP	195	151	340	151	**4,947**	0.004
	BindingDB	1,162	259	5,984	129	22	**290,874**

Target	DrugBank	**2,102**	0.622	0.510	0.108	0.324	0.182
	KEGG	295	**474**	0.418	0.409	0.283	0.348
	TTD	479	198	**940**	0.113	0.434	0.526
	MATADOR	198	194	106	**1,840**	0.262	0.040
	PDSP	47	41	63	38	**145**	0.152
	BindingDB	372	165	494	73	22	**2,048**

Drug-target	DrugBank	**9,886**	0.440	0.200	0.092	0.039	0.149
	KEGG	1,553	**3,532**	0.166	0.254	0.067	0.117
	TTD	1,976	586	**22,791**	0.032	0.042	0.386
	MATADOR	906	898	341	**10,687**	0.023	0.017
	PDSP	384	237	490	247	**11,762**	0.007
	BindingDB	1,471	415	8,804	179	81	**503,378**

^a^The value on the diagonal line of each matrix represents the numbers of drug/target/drug-target pair in each source. The value under the diagonal line of each matrix shows the number of overlap between two sources.

^b^The value above the diagonal line of each matrix shows the proportions of overlap between two sources. The proportion is defined as the number of intersection dividing by the smaller number of the two sources.

After the normalization of drug-target interactome extracted from different sources, we further classified the drugs and the proteins by ATC drug classification system and PANTHER protein class tool, respectively. Supplementary Table S2, Additional file 2 shows the number of drugs in each drug class. Based on the drug's ATC annotation from DrugBank and KEGG, among the 303,018 drugs in WinDTome, 1,856 had ATC codes, of which 1,502 drugs had one ATC code while 354 drugs had multiple ATC codes. Supplementary Table S3, Additional file 2 summarizes the number of protein targets in each protein class in WinDTome. Among the 4,113 protein targets in WinDTome, 2,888 were found in PANTHER. Among them, 2,240 had been annotated to be one single protein class while the rest 648 targets have been annotated to be multiple protein classes. The top 5 protein classes of drug's targets in WinDTome were receptor, transporter, oxidoreductase, kinase, and enzyme modulator.

### Drug-target network and its scoring system

**Figure 2:**
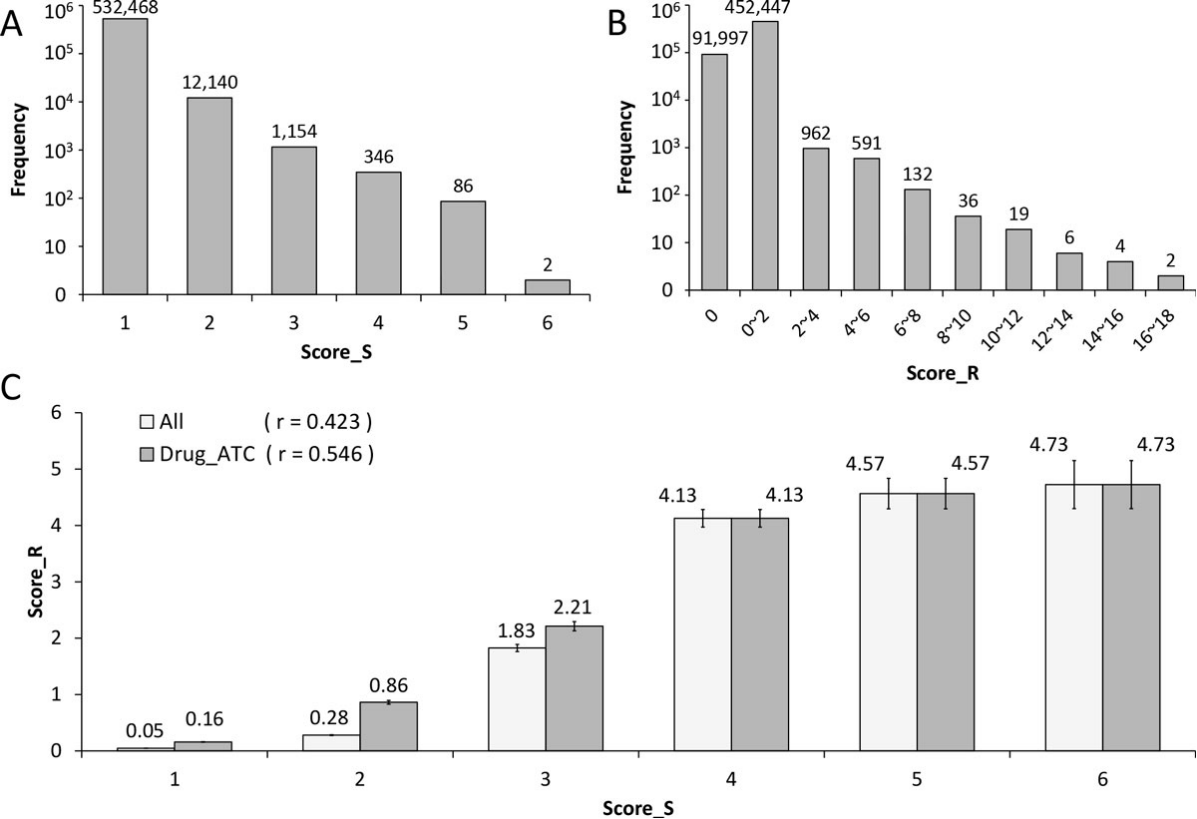
**Distributions and comparison of Score_S and Score_R**. A) The y-axis represents the frequency of drug-target association's Score_S. The label above a bar shows the exact number of the frequency. B) The y-axis represents the frequency of drug-target association's Score_R. The label above a bar shows the exact number of the frequency. C) This bar chart shows the average Score_Rs of all drug-target associations and the associations involving the drugs with ATC codes (represented as "Drug_ATC") in each group of Score_S, respectively. The label above a bar represents the average value of Score_R. The error bar is shown and defined as the standard error of the mean (SEM) of the Score_Rs in each group of Score_S. The r value is the Pearson correlation coefficient between Score_S and Score_R.

To measure the reliability of each drug-target association in WinDTome, we designed two scores (Score_S and Score_R) were designed to. Score_S and Score_R were calculated based on diverse supports: the former was from the number of the concordance of the six sources while the latter was from the summation of the specificities of reference papers. Table 2 shows the numbers of drugs, targets, and drug-target interactions and the average Score_R in each group of Score_S of WinDTome and those in the subset with drug having ATC code. Figure 2A and Figure 2B show the distributions of Score_S and Score_R, respectively. To examine the consistency between the two scores, we performed Pearson correlation analysis. Figure 2C shows the Score_S and Score_R had a positive correlation (r = 0.423). Additionally, the correlation between Score_S and Score_R was 0.546 if we only considered the drug-target associations in which the drugs have ATC codes. Based on the correlation between Score_S and Score_R, we utilized the Score_S ≥ 3 as a threshold for further analysis.

**Table 2:** The distribution of the drugs, targets, their associations, and their Score_R and Score_S scores

	All drugs				Drugs having ATC codes			
	
Score_S^a^	#Drugs	#Targets	#Drug-targets	Score_R^b^	#Drugs	#Targets	#Drug-targets	Score_R^b^
≥ 1	303,018	4,113	546,196	0.059	1,856	2,872	24,381	0.358
≥ 2	8,269	946	13,728	0.534	1,202	589	3,284	1.644
≥ 3	1,034	358	1,588	2.480	753	291	1,256	2.905
≥ 4	367	122	434	4.217	367	122	434	4.217
≥ 5	87	27	88	4.569	87	27	88	4.569
≥ 6	2	1	2	4.725	2	1	2	4.725

^a^The score was calculated based on the occurrence of the drug-target interaction in the six databases.

^b^The score was calculated according to the references of drug-target associations.

### Application of WinDTome for drug repurposing using SCZ as an example

To illustrate the application of WinDTome for drug repurposing, we utilized the SCZ as an example. We collected 41 known SCZ drugs and their 41 target proteins. Among the 41 proteins, 34 existed in the drug-target interactions in the WinDTome with highly confident associations (Score_3 ≥ 3) and were targeted by 224 non-SCZ drugs. These non-SCZ drugs were defined as the first set of potential SCZ drugs. From the 51 proteins, we obtained 563 proteins having interactions with them. Among them, 24 were encoded by SCZ-related genes, from which we obtained 46 non-SCZ drugs that targeted the 7 SCZ-related proteins from WinDTome with highly confident associations (Score_S ≥ 3). We defined them as the second set of potential SCZ drugs. After putting them together, we obtained the 264 unique non-SCZ drugs and 41 unique proteins (34 SCZ drug targets and 7 SCZ-related proteins).

**Figure 3:**
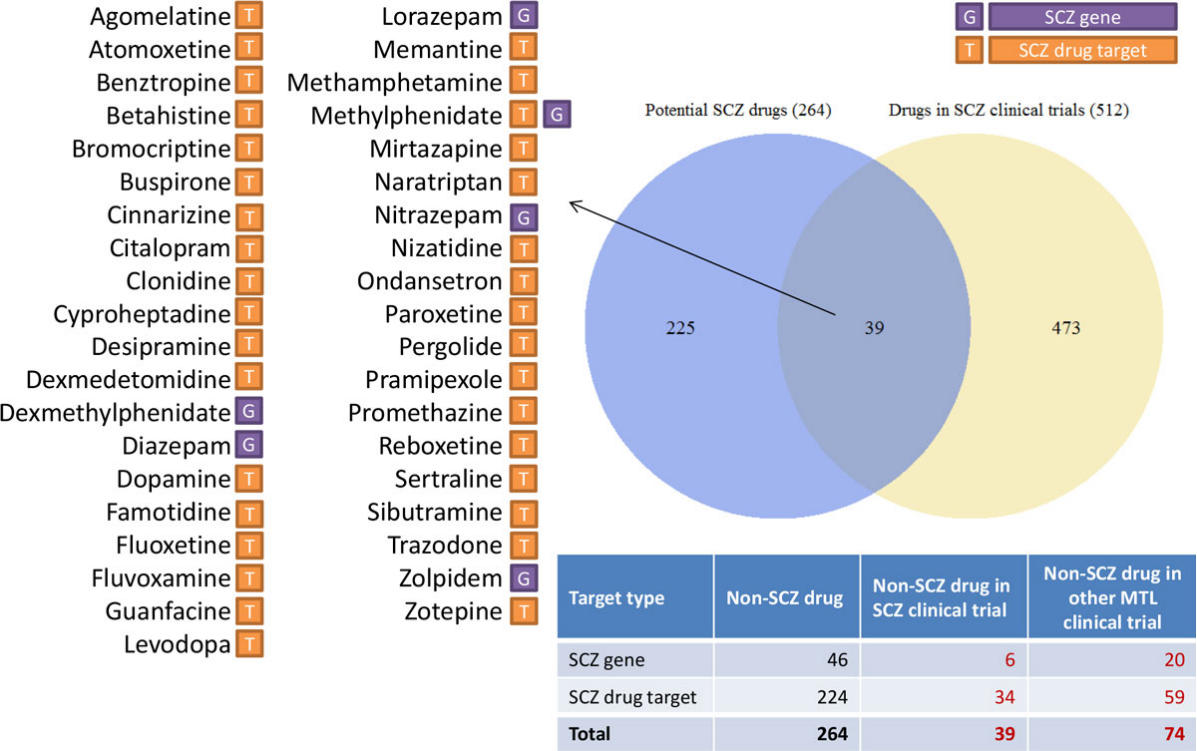
**The 39 potential SCZ drugs and their comparison with other drugs with clinical trials**. Target type means the category of the potential SCZ drug's target. It indicated that inference methods for the potential SCZ drugs. SCZ: schizophrenia; MTL: mental disorders.

To assess if these non-SCZ drugs have potential to treat SCZ, we utilized the clinical trial studies to examine how many drugs have been investigated in schizophrenia or other mental diseases. After querying the 264 non-SCZ drugs against ClinicalTrials.gov, we found that 39 non-SCZ drugs have been investigated in the 82 SCZ-relevant clinical trials and more 74 non-SCZ drugs have been investigated in the 1,831 mental disorders-relevant clinical trials, respectively. There were 512 drugs in the 82 SCZ-relevant clinical trials. Figure 3 summarized that the overlap between the 264 non-SCZ drugs and the drugs in SCZ-relevant clinical trials, listed the 39 drugs and their identification methods (directly from SCZ drug targets, or from target interactors), and also summarized the number of non-SCZ drugs that have been investigated in the other mental diseases in the ClinicalTrials.gov.

**Figure 4:**
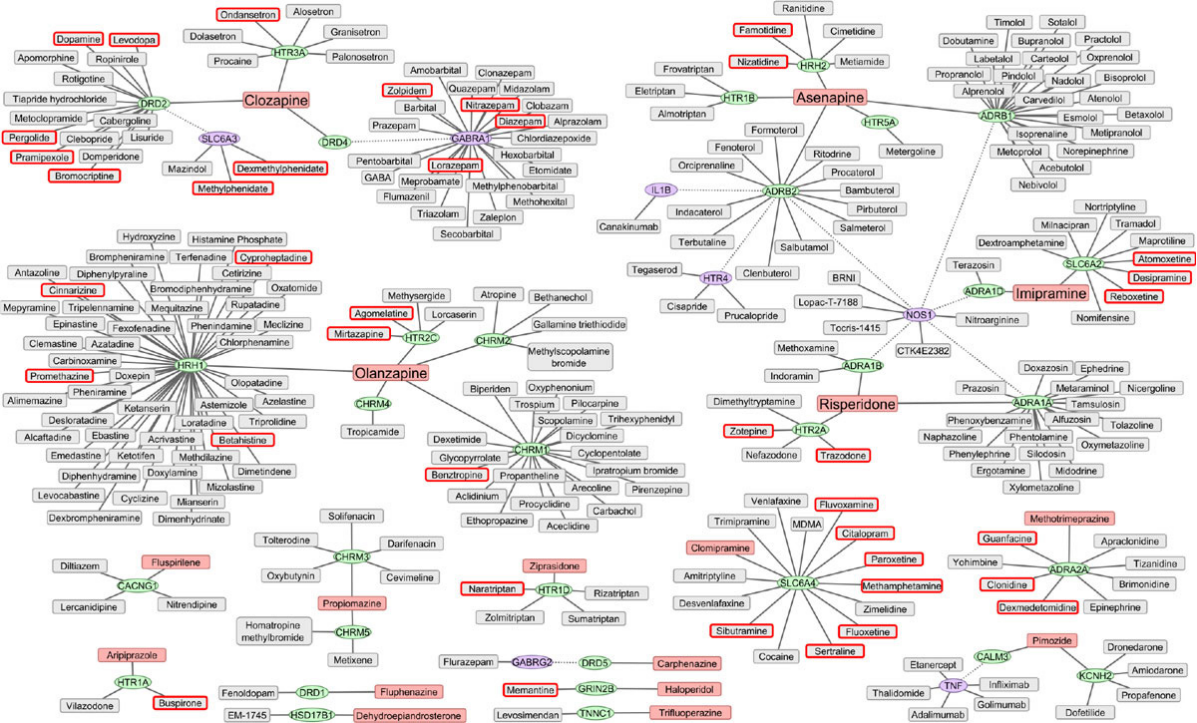
**Expanding drug-target network for predicting potential SCZ drug**. The red rectangular represents the SCZ drug; the blue rectangular represents the potential SCZ drug; the blue rectangular with red border represents the potential SCZ drug related to SCZ-relevant clinical trial; the green ellipse represents the SCZ drug target; the orange ellipse represents the SCZ-related proteins; the solid line between drug and gene represents the highly confident drug-target association; and the dotted line represents the protein-protein interaction.

Table 3 shows the detail number of the clinical trials and the ATC categories of these 39 drugs. The 39 drugs had 24 targets, and Table 4 shows their protein classes. We observed that most of them were either receptors or transporters. Figure 4 represents the expanding SCZ drug-target network plus potential SCZ drugs. To make the figure simple, we did not include the interactions among SCZ drug targets or the interactions among SCZ-related proteins. Additionally, if a potential SCZ drug had associations with multiple SCZ genes/SCZ drug targets, or a SCZ drug target had associations with multiple SCZ drugs, we utilized the associations with the highest Score_S and Score_R to represent the existence of the potential SCZ drugs. The network mainly contained three subnetworks including Olanzapine-centered subnetwork, Clozapine-centered subnetwork, and a subnetwork containing Asenapine, Imipramine, and Risperdone.

**Table 3:** Potential drugs for schizophrenia treatment

Potential SCZ drug	Indication^a^	ATC codes	Number of SCZ clinical trials
Citalopram	Depression	N06AB04; N06AB10	13
Atomoxetine	ADHD^b^	N06BA09	8
Memantine	Parkinson's disease	N06DX01	6
Paroxetine	Depression	N06AB05	6
Sertraline	Depression	N06AB06	5
Fluvoxamine	Depression	N06AB08	4
Lorazepam	Anxiety	N05BA06	4
Ondansetron	Nausea and vomiting	A04AA01	4
Dexmethylphenidate	ADHD	N06BA11	3
Fluoxetine	Depression	N06AB03	3
Methylphenidate	ADHD	N06BA04	3
Benztropine	Parkinson's disease		2
Betahistine	Obesity	N07CA01	2
Clonidine	ADHD	C02AC01; N02CX02; S01EA04; S01EA03	2
Famotidine	Peptic ulcer disease	A02BA03	2
Guanfacine	Hypertension	C02AC02	2
Mirtazapine	Depression	N06AX11	2
Pergolide	Parkinson's disease	N04BC02	2
Reboxetine	Depression	N06AX18	2
Zolpidem	Insomnia	N05CF02	2
Agomelatine	Depression	N06AX22	1
Bromocriptine	Parkinson's disease	G02CB01; N04BC01	1
Buspirone	Anxiety	N05BE01	1
Cinnarizine	Nausea and vomiting	N07CA02	1
Cyproheptadine	Allergies	R06AX02	1
Desipramine	Depression	N06AA01	1
Dexmedetomidine	Anxiety	N05CM18	1
Diazepam	Anxiety	N05BA01; N05BA17	1
Dopamine	Parkinson's disease	C01CA04	1
Levodopa	Parkinson's disease	N04BA01; N04BA04	1
Methamphetamine	ADHD	N06BA03	1
Naratriptan	Migraine headaches	N02CC02	1
Nitrazepam	Insomnia	N05CD02	1
Nizatidine	Peptic ulcer disease	A02BA04	1
Pramipexole	Parkinson's disease	N04BC05	1
Promethazine	Allergies	D04AA10; R06AD02; R06AD05	1
Sibutramine	Obesity	A08AA10	1
Trazodone	Depression	N06AX05	1
Zotepine		N05AX11	1
Total			82

^a^The information of drug indication was obtained from DrugBank and TTD.

^b^ADHD: attention deficit hyperactivity disorder.

**Table 4:** Protein classification of the potential SCZ drug targets

Protein class	Protein	Potential SCZ drugs with clinical trials	
Receptor	ADRA1A	Trazodone	
	
	CHRM1	Benztropine	Desipramine
	
	DRD1	Pergolide	Dopamine
	
	DRD2	Bromocriptine	Pergolide
		Pramipexole	Dopamine
		Buspirone	Levodopa
	
	DRD3	Bromocriptine	Pergolide
		Pramipexole	Dopamine
	
	DRD4	Pramipexole	Levodopa
		Dopamine	
	
	DRD5	Dopamine	
	
	HRH1	Betahistine	Cinnarizine
		Cyproheptadine	Desipramine
		Promethazine	Mirtazapine
	
	HRH2	Famotidine	Nizatidine
	
	HTR1A	Naratriptan	Buspirone
	
	HTR1B	Naratriptan	
	
	HTR1D	Naratriptan	
	
	HTR2A	Mirtazapine	Zotepine
		Trazodone	
	
	HTR2C	Agomelatine	Mirtazapine

Transporter	SLC6A2	Atomoxetine	Desipramine
		Methamphetamine	Reboxetine
		Methylphenidate	Sibutramine
	
	SLC6A3	Dexmethylphenidate	Methylphenidate
	
	SLC6A4	Citalopram	Fluoxetine
		Fluvoxamine	Paroxetine
		Methamphetamine	Sertraline
		Sibutramine	Trazodone

Transporter;	GABRA1	Lorazepam	Diazepam
Receptor		Nitrazepam	Zolpidem
	
	GABRG2	Lorazepam	Diazepam
	
	GRIN2B	Memantine	
	
	HTR3A	Ondansetron	Mirtazapine

(Not available)	ADRA2A	Dexmedetomidine	Clonidine
		Mirtazapine	Guanfacine
	
	ADRA2B	Clonidine	
	
	ADRA2C	Dexmedetomidine	Clonidine

### Evaluation of prediction performance

Based on the data from WinDTome (Score_S ≥ 3), we inferred 264 potential SCZ drugs. According to the clinical trial records in the ClinicalTrials.gov, among them, 39 drugs have been investigated in the schizophrenia treatment and 74 drugs have been investigated in the intervention of other mental diseases. To further evaluate the performance of the prediction, we designed three comparative strategies, including utilizing different score thresholds, single data source, and the data from external integrative database STITCH. Table 5 summarizes the comparative results.

**Table 5:** Potential SCZ drugs identified by using different data sources and threshold

Data source	Threshold	#Potential SCZ drugs	#Drugs in SCZ-CT^a^	#Drugs in MTL-CT^b^
WinDTome	Score_S ≥ 3	264	39 (14.8%)	74 (28.0%)
	Score_S ≥ 2	1,354	76 (5.6%)	102 (7.5%)
	Score_S ≥ 1	37,680	191 (0.5%)	292 (0.8%)

DrugBank		490	45 (9.2%)	87 (17.8%)
KEGG		375	30 (8.0%)	70 (18.7%)
TTD		2,388	27 (1.1%)	62 (2.6%)
MATADOR		374	48 (12.8%)	82 (21.9%)
PDSP		2,001	39 (1.9%)	59 (2.9%)
BindingDB		34,748	41 (0.1%)	98 (0.3%)

STITCH	Combined score ≥ 0.7	32,282	100 (0.3%)	241 (0.7%)

^a^SCZ-CT represents the schizophrenia-relevant clinical trials.

^b^MTL-CT represents the mental disorders-relevant clinical trials.

Using the data in WinDTome whose Score_S no less than 2 or 1, we performed the inference process as the data with Score_S were no less than 3, respectively, and calculated the proportion of the potential SCZ drugs that have been investigated to treat SCZ in the clinical trials. We observed that the proportion of the potential SCZ drugs found in SCZ-relevant clinical trials based on Score_S ≥ 3 as a threshold (14.8%) was higher than that of the threshold Score_S ≥ 2 (5.6%) and the threshold Score_S ≥ 1 (0.5%), respectively. Similarly, the proportion of the potential SCZ drugs found in other mental disorders, according to clinical trials using the threshold of Score_S ≥ 3 was higher than those based on the other two thresholds (28.0% vs. 7.5% and 0.8%).

We repeated the inference process by using the data from each data source for the WinDTome creation. Some performance based on single data sources were higher than those based WinDTome with Score_S ≥ 2 or Score_S ≥ 1; however, none of them is higher than those of using WinDTome with Score_S ≥ 3. It implies that the scoring system we used is promising in conducting network pharmacology studies, and the recommending cutoff of Score_S is reasonable.

Finally, we performed the prediction using the data from the STITCH for potential SCZ drugs. We extracted 455,430 high confident (combined score ≥ 0.7) drug-target associations among 199,133 drugs and 9,379 unique human proteins from STITCH. We obtained 29,405 non-SCZ drugs having associations with known SCZ drug targets and 5,447 non-SCZ drugs targeting the 51 SCZ-related proteins. In total, we obtained 32,282 unique non-SCZ drugs. In the ClinicalTrials.gov, we found 100 out of the 32,282 potential SCZ drugs in SCZ-relevant clinical trials and 241 potential SCZ drugs in other mental disorders-relevant clinical trials.

In summary, the comparative results indicated that the scoring strategy designed in this study provides an assessment of the reliability of the drug-target associations, and WinDTome provides weighted drug-target associations.

## Discussion

In this study, we built a weighted and integrated drug-target interactome (WinDTome) to provide a fundamental for computational drug repurposing. In WinDTome, we assigned two scores for each drug-target association, which provides an assessment of the reliability of the drug-target associations. Starting from the drug-target associations in WinDTome and SCZ known drugs and their targets, we inferred the potential drugs for SCZ treatment. The illustration indicates that weighted WinDTome provides one promising source for drug repurposing.

We inferred the 264 potential candidate drugs that could be used to treat SCZ. Among them, 39 potential drugs have been investigated in schizophrenia according to the clinical trial records from the clinicaltrials.gov. We further checked the indications of these drugs in DrugBank and TTD and then categorized them into six indication groups: depression, anxiety, Parkinson's disease, insomnia, attention deficit hyperactivity disorder (ADHD), and others (Table 3). Most of them are related to mental disorders. Among the 264 potential SCZ drugs, 113 have been investigated in the mental disorders. Among them, the drugs treating depression have the most number of clinical trials. It is not surprising that the four potential SCZ drugs - citalopram, paroxetine, sertraline, and fluvoxamine - might have relevant mechanisms and resembling biological pathways with existing SCZ drugs because depression is one of the negative symptoms of schizophrenia. In addition to the positive and negative symptoms, SCZ patients are highly inclined to have comorbidities such as anxiety disorders, depression, and diabetes [39,40]. Interestingly, in the study "NCT01401491" (an identifier in the ClinicalTrials.gov) [41], Fluvoxamine was combined with clozapine to treat schizophrenia patients, and the clinical improvement was observed in the investigators' patients. The investigators found that fluvoxamine could reduce the clozapine-induced side effects of metabolic disturbance and obesity. The use of fluvoxamine as a co-administration for the treatment of cognitive impairments in patients with schizophrenia has been reported [42,43], and the potential of Fluvoxamine for treating schizophrenia was also proposed [44]. In the 39 potential SCZ drugs with clinical evidences, we also found that betahistine and sibutramine are used to treat obesity. Betahistine, a nervous system drug, and Sibutramine, a centrally acting antiobesity product, were proposed to be co-administrations for treating schizophrenia patients, respectively [45,46]. These findings were consistent to the a previous study mentioning that prescribed co-administrations of antipsychotics drugs and atypical substances that have been increasing for treating SCZ patients along with various comorbidities [47]. Moreover, both Parkinson's disease and schizophrenia are related to the dopaminergic system [48]. Thus, the four Parkinson's disease drugs - benztropine, pergolide, levodopa, and pramipexole - may also have potential for schizophrenia treatment [49-51].

The inference strategy for drug repurposing started with the known drugs that have been used to treat SCZ and their targets. The underlying assumption is that the potential drugs for a given disease should have similar molecular mechanisms or actions to the known drugs. Therefore, this strategy provides a methodology for any diseases with effective medicines. To examine the robustness of the network-based drug repurposing methodology in this study, we further performed the similar inference of drug repurposing using colon cancer as another example. We manually collected 12 approved colon cancer drugs from DrugBank (aflibercept, bevacizumab, capecitabine, cetuximab, fluorouracil, irinotecan, leucovorin, oxaliplatin, panitumumab, raltitrexed, regorafenib, and trimetrexate) and 38 drug targets (human genes), and the colon cancer associated genes from Cancer Gene Census [52]. By implementing the same processes of network-based inference, we obtained 26 potential colon cancer drugs with high confident associations with known colon cancer drug targets. Among them, 16 were found in clinical trials whose condition was "colon cancer." The numbers of these 16 drugs' colon cancer relevant trials, as well as the drugs' indications and ATC codes, are provided in Supplementary Table S4, Additional file 2. In addition, the protein classes of these 16 potential colon cancer drug targets are provided in Supplementary Table S5, Additional file 2. In summary, this preliminary result further suggested that the network-based inference for drug repurposing by using the drug-target associations in WinDTome is promising.

The inference of drug repurposing mainly relies on the drug-target interactome. In this study, based on the analysis of Score_S and Score_R distributions, we mainly employed highly confident drug-target associations (Score_S ≥ 3) to identify the candidates. However, some potential candidates will be missed compared to the inference based on the concept of off-target and drug target similarity. Thus, integrating the other data, such as drug structure similarity, off-target information, and target similarity, might improve further the performance. Therefore, it still is necessary to develop a more comprehensive scoring system for the drug-target associations. Additionally, in this study, we mainly employed the direct protein-protein interactions (PPIs) of the drug targets and disease genes, which is restricted to the network environment information. In the future, we will consider the effects of drug targets on the disease genes in the context of PPI networks.

## Conclusion

In this study, we generated a weighted and integrated drug-target interactome (WinDTome), which provides a reliable source of drug-target interactome. We utilized the WinDTome along with a network pharmacology approach for drug repurposing for SCZ as an example. Eventually, we successfully obtained 264 potential SCZ drugs, including 39 drugs that have been found in existing SCZ-relevant clinical trials and 74 drugs that have been investigated in other mental disorders. We further compared the results with the inference using different thresholds, single data source, and the data from the commonly used database STITCH. The comparative result further demonstrated that the WinDTome is promising for further systems pharmacology study.

## Competing interests

The authors declare that they have no competing interests.

## Authors' contributions

LH and JS collected data for the study. LH and JS performed data analysis. JS and HX conceived and designed the study. LH and JS wrote the manuscript. ES, WZ, JS, ZZ revised the manuscript.

## Declaration

The publication funding came from a 2013 NARSAD Young Investigator Award to Dr. Jingchun Sun.

This article has been published as part of *BMC Systems Biology *Volume 9 Supplement 4, 2015: Selected articles from The International Conference on Intelligent Biology and Medicine (ICIBM) 2014: Systems Biology. The full contents of the supplement are available online at http://www.biomedcentral.com/bmcsystbiol/supplements/9/S4.
